# Incident cases characterization and deep sequencing provide new insight into multiplicity of infection and HIV evolution in very early acute infection

**DOI:** 10.1186/1742-4690-9-S2-P143

**Published:** 2012-09-13

**Authors:** G Kijak, E Sanders-Buell, M Rolland, H Li, A Bates, M Bose, A O'Sullivan, L Eller, R O'Connell, G Shaw, N Michael, J Kim, M Robb, S Tovanabutra

**Affiliations:** 1 U.S. Military HIV Research Program/Henry M. Jackson Foundation, Silver Spring, MD, USA; 2 Perelman School of Medicine, University of Pennsylvania, Philadelphia, PA, USA; 3 U.S. Military HIV Research Program/WRAIR, Silver Spring, MD, USA

## Background

RV217/ECHO aims at capturing HIV-1 incident cases during the very earliest stages of acute HIV-1 infection(AHI). Limitations in single genome amplification(SGA) sampling depth have the potential to confound the study of multiplicity of infection and viral evolution during AHI. Here we combined SGA and deep, next-generation sequencing(NGS) to investigate the prevalence of minor viral variants during pre-peak viremia and characterize subsequent viral evolution.

## Methods

A male CSW from Thailand with documented nucleic acid testing(NAT)-conversion and seroconversion, and with measured viremia peak, was studied. Plasma viruses from pre-peak viremia(9 days after last negative NAT and 2 days after first positive NAT; pVL=891,251), immediate post-peak viremia(38 days after last negative NAT; pVL=512,861), and 6 months post-infection(pVL=181,970) were characterized by SGA and targeted NGS. A library prepared from 1,500 PCR-equivalent copies was analyzed using IonTorrent(LifeTechnologies).Reading coverage was>20K and the experimentally-measured error rate was<0.0028/base.

## Results

Based on 47 env SGA sequences from pre-peak viremia, the infection was initiated by a single T/F virus (mean env pair-wise nt diversity:0.047%).A second variant, highly similar but distinguishable from the T/F (env nt distance:2.4%;env AA distance:3.4%), was detected during immediate post-peak viremia(18/56 SGA sequences). By 6 months post-infection, the viral population was dominated by the second variant along with numerous recombinants.

NGS analysis of multiple, independent subgenomic areas with concentrated sequence variation, revealed that the second variant was already established at 1% at pre-peak viremia. Outgrowth of the second variant, along with its derivatives, was confirmed by longitudinal NGS.

## Conclusion

Supplementing SGA analysis with NGS allowed us to demonstrate the presence of two T/F viruses at pre-peak viremia with substantial frequency differences, in the study participant. Due to its implications, the prevalence of this phenomenon needs to be further explored. The application of NGS to the study of HIV-1 evolution during AHI promises to foster the advancement of vaccine development.

